# *Coffea arabica* Seed Extract Stimulate the Cellular Immune Function and Cyclophosphamide-induced Immunosuppression in Mice 

**Published:** 2013

**Authors:** Mohammad Rafiul Haque, Shahid Hussain Ansari, Azhar Rashikh

**Affiliations:** a *Department of Pharmacognosy and Phytochemistry, Jamia Hamdard, Hamdard Nagar, New Delhi 62, India.*; b *Department of Pharmacology, Faculty of Pharmacy, Jamia Hamdard, Hamdard Nagar, New Delhi 62, India. *

**Keywords:** *Coffea Arabica*, Hemagglutination titre, Delayed type hypersensitivity

## Abstract

In this study, we investigate the immunostimulatory effects of alcoholic extract of the coffee seed on cell-mediated immune response and cyclophosphamide-induced (CP) immunosuppressed mice. The assessment of cellular immune function was carried out by the measurement of delayed-type hypersensitivity (DTH) response. According to the literature survey, cyclophosphamide has only suppressing effect on the lymphoid organ, white blood cell (WBC) and other parts of humoral immunity. Humoral immunity was assessed by the hemagglutination antibody titre. Mice were treated with three doses of extract (50, 150 and 250 mg/Kg body weight per os). Relative organ weight and WBC counts were also studied in these animals. At doses of 50 and 150, a significant increase (p < 0.05) in relative organ weight of spleen and thymus was observed but there was no effect on kidney and liver weights. WBC counts was also increased significantly (p < 0.001) in all doses of the plant extract. *Coffea arabica *extract elicited a significant (p < 0.001) increase in the DTH response at doses of 50 and 150 mg/Kg, but the change at higher dose of 250 mg/Kg was not statistically significant. In the HT test, plant extract also showed modulatory effect at all doses groups. Over all, coffee seed showed the stimulatory effect on cellular immune function and cyclophosphamide induced immunosuppression in mice.

## Introduction

The immune system consists of various cell types, some of which have opposing functions; for example, T helper type 1 (TH1) cells promote, whereas TH2 cells suppress, delayed-type hypersensitivity (1). The body’s immunity has been shown to be suppressed in several diseases like AIDS and cancer, and the use of immunostimulatory agents can solve these problems to a great extent. Immunomodulation is a procedure that enhances the immune function of the organism by interfering with its function. If drug treatment enhances immune reactions, it is termed an immunostimulatory drug, which primarily implies the stimulation of nonspecific system, that is, granulocytes, macrophages, complement, certain T lymphocytes, and different effectors’ substances (2). The greatest disadvantage of using synthetic immunomodulatory agents is their side effects, viz., neutropenia, anorexia, and proteinemia (3); therefore, plant-based immunomodulators can thus become a better choice for chemotherapy (4-7). Various plant extracts have immunostimulatory activity as evidenced by increased proliferation of lymphocytes and production of interleukin-2 (8). The various plant-derived include alkaloids, quinones, terpenoids, phenol carboxylic acids, and high-molecular mass-compounds such as polysaccharides and glycoprotein’s natural products have immunostimulatory activity (9). Medicinal plants serve as therapeutic alternatives, safer choices, or in some cases, as the only effective treatment. A large number of these plants and their isolated constituent have shown beneficial therapeutic effects, including antioxidant, anti-inflammatory, anticancer, antimicrobial, and immunomodulatory effects (10). In present study, we have been undertaken to explore the immunostimulatory effect of coffee beans in animal model.

## Experimental

*Animals*

The study was approved by the Institutional Animal Ethics Committee (IAEC) of Hamdard University, New Delhi, which is registered with “Committee for the Purpose of Control and Supervision of Experiments on Animals” (CPCSEA) in the use of animals for scientific research. Animals (CPCSEA), Government of India, India (Registration No. 173/CPCSEA, 28 Jan 2000). Female albino mice (Swiss) of weighing 31-35 g, were procured from the Central Animal House Facility, Hamdard University, New Delhi, and acclimatized under standard laboratory conditions at 25 ± 2°C, and relative humidity (50% ± 15%) and normal. The animals were kept in polypropylene cages under standard laboratory conditions (12 h light and 12 h dark cycle) and had free access to tap water *ad libitum*.

*Chemicals*

Cyclophosphamide was purchased from Sigma (Aldrich). Sheep red blood cell (SRBC) was obtained from slaughter house, Pahar Ganj, New Delhi, India.

*Plant material and extract preparation*

Fresh dried coffee bean was collected from Yucca Enterprises, Mumbai at the month of July and authenticated by Dr. H. B. Singh from National Institute of Science Communication and Information Resources (NISCAIR), New Delhi. Voucher specimen and identification certificate reference number NISCAIR/RHMD/Consult/2008-08/966/150 were obtained and kept in the department for future reference. The dried seeds were powdered (sieve number 80) and kept separately. Heavy metal (arsenic, lead, cadmium and mercury), Aflatoxins (B1, B2, G1, and G2) and pesticide residues such as o, p-DDD, p, p-DDD, o p-DDE, p, p-DDE, o, p-DDT, p, p-DDT, Endosulfan, *α*-HCH, *β*-HCH, *γ*-HCH, *δ*-HCH were not detected in the coffee seed powder. Alcoholic extracts (8.678 w/w) of coffee bean were prepared using soxhlet apparatus.

*Chemical analysis of extract*

The alcoholic extract showed the presence of flavonoid, phenolics, glycoside, saponins, alkaloid, and polysaccharide when subjected to qualitative chemical tests.

**Figure 1:**
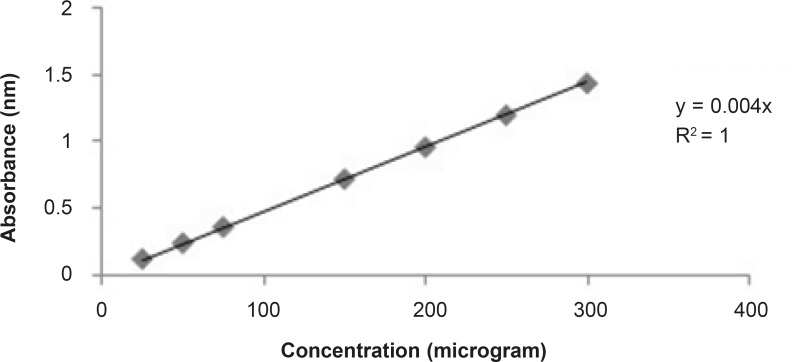
Graph of standard curve of Gallic acid for total phenolic contents

**Figure 2:**
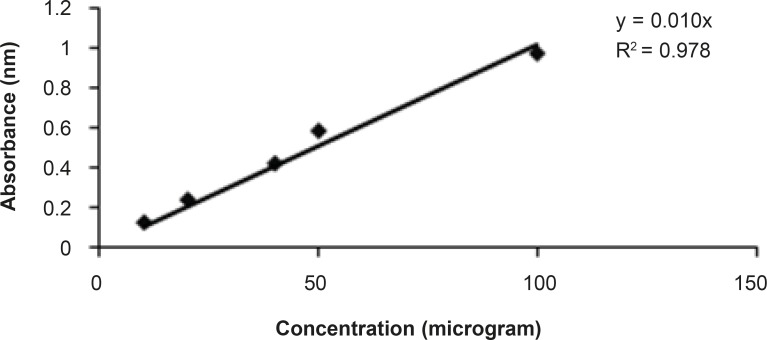
Graph of standard curve of quercetin for total flavonoid contents.

The total phenolic contents estimated were done according to standard published method Folin Ciocalteu reagent (11). Aluminium chloride colorimetric method was used for flavonoid determination (12). Total phenolic and flavonoid contents were found about 13% and 3% respectively in the bean of Coffea arabica. The standards graph of gallic acid and quercetin for total phenolic and flavonoid contents are shown in Figures 1 and 2, respectively.

**Figure 3:**
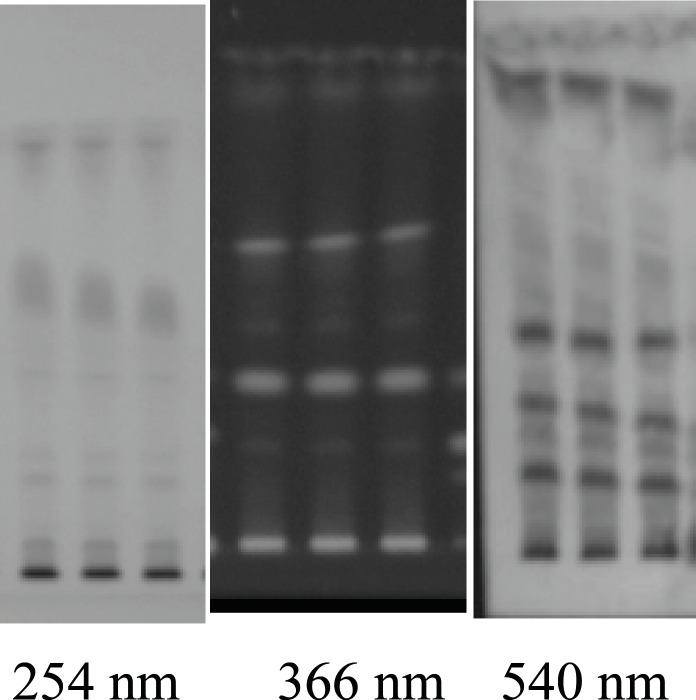
TLC Plate developed at 254 366 nm and A.S reagent (540 nm) in triplet form

For HPTLC fingerprints, the alcohol extracts were applied on prepared precoated silica gel 60 F254 TLC plate (E. Merck) as absorbent and developed the plate using solvent systems toluene: ethyl acetate: methanol: formic acid ( 2.5: 1.5 : 0.8 : 0.1). After developing, the plates were dried and the color spots were observed at UV-254, and UV-366 nm and anisaldehyde -sulphuric acid spraying reagent. The *R*f values of different spots were calculated. The fingerprint chromatograms are shown in Figure 3. Details of the fingerprint analysis are given in (Table 1). Heavy metals analysis (Hg, As, Cd and Pb), Pesticide residues and aflatoxin were also absent in the drug.

**Table 1:** HPTLC fingerprints of alcoholic extract

**Solvent system**		**Toluene: Ethyl Acetate: Methanol: Formic Acid** **(2.5 : 1.5 : 0.8 : 0.1)**
**Rf Values**	UV 254 nm	0.06, 0.10, 0.23, 0.29, 0.34, 0.47, 0.52, 0.64, 0.72, 0.87
	UV 366 nm	0.09, 0.29, 0.39, 0.49, 0.60, 0.73, 0.83, 0.87
	A.S.Reagent	0.9, 0.10, 0.13, 0.33, 0.34, 0.37, 0.39, 0.45, 0.46, 0.57, 0.60, 0.62, 0.65, 0.73, 0.75, 0.82, 0.86, 0.88, 0.95

*Drugs*

Accurately weighed quantities of the ethanolic extract were suspended in 1% sodium carboxymethylcellulose (SCMC) to prepare a suitable dosage form. Cyclophosphamide was used as a standard immunosuppressant. Sheep red blood cells (SRBC) were used as an antigen at the concentration of 20% for immunization and 1% for challenge.

*Relative organ weight*

 Animals were divided into four groups (I–V), each of which comprised a minimum of six animals. Group I (control) received normal saline, group II (CP) were injected with a single dose of CP on the twelf^th^ day of initiation of experiment, groups III, IV and V (plant extract + CP) were given plant extract treatment for 14 days with a single injection of CP on twelf^th^ day. The animals were sacrificed by cervical dislocation 24 h after the last dose. Relative organ weight (organ weight/100 g of body weight) of kidney, liver, spleen and thymus were determined for each animal Raisuddin *et al. *(13).

*Total leucocytes count (WBC count)*

For WBC count, we adopted the method described by Manjarekar *et al *(14). Animals were divided into five groups of six animals. Group I (control group) and group II (cyclophosphamide-treated control) received the vehicle (1% SCMC, p.o.) for a period of 13 days. Groups III-V were given the test extracts (50-250 mg/Kg, p.o.) daily for 13 days. The animals of groups II-V were injected with cyclophosphamide (30 mg/Kg, IP) on the 11^th^, 12^th^ and 13^th^ days, 1 h after the administration of the respective treatment. Blood samples were collected one day before the experiment (day 0) and on the 14^th^ day and the total white blood cells (WBC) count was determined using a haemocytometer.

*Haemagglutinin titre (HT) and delayed type hypersensitivity (DTH) response*

It was performed using procedure of Puri *et al. *(15). Animals were divided into five groups of six animals each. Group I (control group) and group II (cyclophosphamide-treated control) received the vehicle (1% SCMC, p.o.) for a period of 7 days. Groups III-V were given the test extracts (50-800 mg/Kg, p.o.) daily for 7 days. The animals of groups II-V were injected with cyclophosphamide (50 mg/Kg, IP) on the 4^th^, 5^th^ and 6^th^ day, 1 h after the administration of the respective treatment. The animals were immunised by injecting 0.1 mL of 20% of fresh SRBC suspension intraperitoneally on day 0. Blood samples were collected in micro centrifuge tubes from individual animals by retro-orbital plexus on the 7^th^ day and serum was separated. Antibody levels were determined by haemagglutination technique. Briefly, equal volumes of individual serum samples of each group were pooled. Two-fold dilutions of pooled serum samples were made in 25 μL volumes of normal saline in micro titration plate and to that was added 25 μL of 1% suspension of sheep red blood cells in saline. After mixing, the plates were incubated at room temperature for 1 h and examined for haemagglutination under microscope. The reciprocal of the highest dilution of the test serum giving agglutination was taken as the antibody titre.

The thickness of the right hind footpad was measured using Plethysmometer on the 7^th^ day. The mice were then challenged by injecting 20 μL of 1% SRBCs in right hind footpad and after 24 h of this challenge, the foot thickness was measured again. The pre- and post-challenge differences in the thickness of footpad were expressed in mm and taken as a measure of DTH.

*Liver functions enzyme evaluations*

Serum Oxaloacetate transaminase (SGOT) and glutamate pyruvate transaminase (SGPT) were estimated by the procedure of kit method. Alkaline phosphatase (ALP) was estimated using phenyl phosphate as substrate; 0.5 mL substrate was incubated at 37°C for 3 min followed by the addition of 0.1 mL serum and again incubated for 30 min at 37°C. Then, dinitrophenylhydrazine (DNPH) (5 mL) was added to the mixture. The reaction was stopped by the addition of 5 mL 0.4M NaOH. Optical density was measured at 512 nm (kit method).

*Statistical analysis*

Data were statistically analyzed using student’s T-test to determine significant differences in data of various groups; p-values less than 5% were considered significant. The value is expressed as mean ± SEM.

## Results

*Effect of plant extracts on lymphoid organ weight*

The extract did not alter the relative weight of kidney and liver in test dose, however at significant increase, changes were observed in the relative weight of spleen at dose of 150 mg/Kg (p < 0.05) and thymus at dose of 50 and 150 mg/Kg (p < 0.05) as compared with group II (Table 2).

**Table 2:** Effect of alcoholic extract of *Coffea arabica on *relative organ weight

**Group**	**Treatments**	**Dose **(**mg/kg**)	**kidney**	**Relative organ weight (g) means ± SEM **		
				**spleen**	**Liver**	**Thymus**
1	Control	-	1.085 ± 0.080	0.79 ± 0.02	5.45 ± 0.22	0.156 ± 0.12
11	CP	30	1.055 ± 0.028	0.20 ± 0.142**^a^	5.43 ± 0.12	0.083 ± 0.04*^,a^
111	Alcoholic extract + CP	50	1.065 ± 0.028	0 .45 ± 0.123	5.22. ± 0.102	0.103 ± 0.02* ^b^
1V	Alcoholic extract + CP	150	1.095 ± 0.016	0.5 ± 0.098*^, a, b^	5.02 ± 0.02	0.143 ± 0.02* ^b^
V	Alcoholic extract + CP	250	1.090 ± 0.087	0. 35 ± 0.088	4.92. ± 0.32	0.93 ± 0.03

n = 6 mice per group. *p < 0.05, **p < 0.01, ***p < 0.001. ^a^ Groups II-V were compared with group I. ^b^ Groups II-V were compared with group II

*Effect of plant extracts on total leucocytes*

A significant (p < 0.001) reduction in WBC count was observed in animals treated with cyclophosphamide alone (group II) as compared with the control group (group I). The ethanolic extract of *C. arabica *increased the levels of WBC at the dose levels of 50 mg/Kg (p < 0.01), 150 mg/Kg (p < 0.001) and 250 mg/Kg (p < 0.01) as compared with cyclophosphamide-treated control group (group II). It was observed that the extract at doses of 150 and 250 mg/Kg restored the levels of WBC back to normal (Table 3).

**Table 3:** Effect of alcoholic extract of *Coffea arabica *total leucocytes counts

**Group**	**Treatments**	**Dose **(**mg/kg**)	**TLC Count (10** ^3^ **/mm**^3^ **) Means ± SEM**	
			**Day 0**	**Day 14**
1	Control	-	5.85 ± 0.088	10.99 ± 0.260
11	CP	30	5.95 ± 0.088	3.80 ± 0.142 ***^,a^
111	Alcoholic extract + CP	50	5.65 ± 0.088	9.25 ± 0.123**^,b^
1V	Alcoholic extract + CP	150	6.95 ± 0.088	11.25 ± 0.098***^,b^
V	Alcoholic extract + CP	250	6.90 ± 0.088	11. 95 ± 0.088***^,b^

n = 6 mice per group. *p < 0.05, **p < 0.01, ***p < 0.001.^ a ^Groups II-V were compared with group I.^ b^ Groups III-V were compared with group II

*Effect of plant extracts on humoral immunity*

A significant (p < 0.001) reduction in antibody titer count was observed in animals treated with cyclophosphamide alone (group II) as compared with the control group (group I). Antibody titer response was significant (p < 0.001) increase observed at all doses as compared with group II (Table 4).

**Table 4:** Effect of alcoholic extract of *Coffea arabica on *antibody titer

**Group**	**Treatments**	**Dose **(**mg/kg**)	**Antibody titer Means ± SEM**
1	Control	-	159.05 ± 48
11	CP	50	19.95 ± 0.088***,^a^
111	Alcoholic extract + CP	50	109.65 ±10.088***,^b^
1V	Alcoholic extract + CP	150	175.00 ±32.88***,^b^
V	Alcoholic extract + CP	250	1 65.00 ± 56.08 ***,^b^

n = 6 mice per group. *p < 0.05, **p < 0.01, ***p < 0.001. ^a^ Groups II-V were compared with group I. ^b^ Groups III-V were compared with group II

*Effect of plant extracts on delayed type hypersensitivity response*

The animals treated with the extract showed a significant change in the DTH response at all dose levels as compared with group I. A significant increase in DTH response was also observed at doses of 50 mg/Kg (p < 0.01) and 150 mg/Kg (p < 0.001) as compared with group II. The higher dose (250 mg/Kg) was not statistically significant as compared with group 11 (Table 5).

**Table 5:** Effect of alcoholic extract of *Coffea arabica on *DTH response

**Group**	**Treatments**	**Dose **(**mg/kg**)	**DTH response (mm) mean** **paw oedema ± SEM**
1	Control	-	0 .35 ± 48
11	CP	50	1.05 ± 0.088***,^a^
111	Alcoholic extract + CP	50	1.15 ±10.088**^, a, b^
1V	Alcoholic extract + CP	150	1.75 ±32.88***^, a, b^
V	Alcoholic extract + CP	250	0 .75 ± 56.08*^,a^

n = 6 mice per group. *p < 0.05, **p < 0.01, ***p < 0.001 ^a ^Groups II-V were compared with group I. ^b^ Groups III-V were compared with group II

*Effect of plant extracts on liver enzymes*

There was no significant elevation in the level of SGOT, SGPT and ALP as the result of C. arabica treatment at any dose used in this study (p < 0.05).

## Discussion

Coffee bean has been reported as antioxidant, anti-obesity and hepatoprotective activity (16, 17). In this study, coffee seed showed stimulatory effect on the immune functions.

Plant extract at doses of 50 and 150 mg/Kg increased the weight of spleen and thymus, but had not any significant result obtained in the case of liver and kidney weight. The increase in thymus and spleen weight was accompanied by increase in its cell counts. In the case of thymus, this may be partly due to the stimulatory effect of plant extract on the lymphocytes and bone marrow haematopoietic cells, which ultimately home in the thymus. Coffee seed extract also did not alter the level of liver functions enzyme as the weight of liver did not get any significant results.

Cyclophosphamide acts on both cyclic and intermitotic cells, resulting in general depletion (suppression) of immune-competent cells. Cyclophosphamide (CP) is one of the most popular alkylating anticancer drugs which produced toxic side effects including immunotoxicity, hematotoxicity and mutagenicity (18, 19). The high degree of cell proliferation changes the bone marrow from a sensitive target particularly to cytotoxic drugs. In fact, bone marrow is highly affected during any immunosuppression therapy with this class of drugs. Damaged or loss of stem cells of the bone marrow unable to regenerate new blood cells result in thrombocytopenia and leucopoenia (20). The administration of *Coffea arabica *seed extract was found to increase the total WBC count, which was lowered by cyclophosphamide, a cytotoxic drug that indicates its stimulatory effect on haematopoietic stem cell of the bone marrow.

Antibody production of T-dependent antigen of sheep red blood cell (SRBC) requires co-operation of T- and B-lymphocytes and macrophages (21). Cyclophosphamide has a particularly intense effect on short-lived lymphocytes known to include a great proportion of B-cells. In HT test, the plant showed an increase response in all doses. The high values of haemagglutinating titre (HT) obtained in case of *Coffea arabica *have indicated that immunostimulation was achieved through the humoral immunity. This activity could be due to the presence of flavonoids which augment the humoral response, by stimulating the macrophage and B-lymphocyte subsets involved in antibody synthesis (22).

In DTH test, the DTH response, which directly correlates with the cell mediated immunity (CMI), was found to be significant at dose of (50 and 150 mg/Kg) tested in the extract. The mechanism of action behind this elevate of DTH during the CMI response could be due to the sensitized T-lymphocytes. When challenged by the antigen, they were converted to lymphoblast and secrete a variety of molecules including proinflammatory lymphocytes, attracting more scavenger cells to the site of reaction. The increasing of DTH response indicate the stimulatory effect of the plant which has occurred on the lymphocytes and accessory cell types required for the expression of this reaction.

On the basis of phytochemical investigation, the main chemical constituents of coffee bean are phenolic, flavonoid, alkaloid, polysaccharides, glycoside and terpenoid compound. TLC fingerprints also showed the NO. of chemical constituent present in the extract. We also estimated the phenolics (13%) and flavonoids (3%) content in the coffee seed. Recent report indicates that several types of flavonols stimulate the human peripheral blood leukocyte proliferation. They significantly increase the activity of helper T-cells, cytokines, interleukin 2, interferon and macrophages and are thereby useful in the treatment of various diseases caused by immune dysfunction (23). Plant containing alkaloids, polysaccharides and terpenoids possesses strong immunostimulatory effect. The immunostimulatory effects of coffee seed may be produced due to the presence of terpenoid and high molecular mass compound such as polysaccharide and glycoprotein. The Alkaloids from various plants have been shown to stimulate the lymphocyte proliferation (24). The Humoural antibodies that are capable of killing free tumour cells in blood and in serosal cavities have been suggested to play an incredibly important role in cancer (25). Coffee seed can be used for the treatment of cancer due to its stimulatory effect on haemagglutinating antibody titre. The obtained result shows that *Coffea arabica *given to the immunosuppressed mice increases the number of antibodies in their serum. According to the finding of this study, coffee seed extract showed an immunostimulatory effect on cellular immune function and cyclophosphamide-induce immunosuppression. In present study, the extract of coffee seed also reflects the direction of developing safer strategies for cancer treatment.

The seeds of *C. arabica *are known for their pharmacological activity and in this manuscript, it has been shown that the extracts can be used as an effective immunostimulatory effect. From these results, we conclude that coffee seed contains phenolic, flavonoid, alkaloid, polysaccharides and terpenoid compounds having a nontoxic immunostimulatory effect. Thus, the stimulatory effect produced by alcoholic extract of *Coffea arabica *in cellular immune functions and cyclophosphamide-induced immunosuppression may be due to the cell-mediated and humoral antibody-mediated activation of T and B cells. It can therefore be concluded that *Coffea arabica *is a potential immunostimulant against the cytotoxic drugs and can be used as a complimentary therapeutic agent.
